# A case of mammary-type myofibroblastoma of the inguinal region

**DOI:** 10.1016/j.ijscr.2018.11.048

**Published:** 2018-11-23

**Authors:** Atsushi Ishihara, Takeo Yasuda, Yukari Sakae, Masayuki Sakae, Tooru Hamada, Hideki Tsukazaki, Takashi Tsukazaki, Masaru Furumoto

**Affiliations:** a Department of Surgery, Tsukazaki Hospital, Himeji, 671-1227, Japan; b Department of Urology, Tsukazaki Hospital, Himeji, 671-1227, Japan; c Department of Pathology, Tsukazaki Hospital, Himeji, 671-1227, Japan

**Keywords:** Mammary-type myofibroblastoma, Inguinal tumor, Case report

## Abstract

**Introduction:**

Myofibroblastoma is usually occurred in the breast and extra-mammary disease is rare.

**Presentation of case:**

A 38-year-old man was admitted to our hospital for further examination of the left inguinal tumour, present and enlarging for 16 months. The tumor was 50 mm in diameter, well-circumscribed, firm, and painless. Ultrasonogaphy, computed tomography(CT) and magnetic resonance imaging (MRI) could not provide the definitive diagnosis. Surgical exploration confirmed a 50 mm tumour with a clear surface with a thin capsule. Complete excision was achieved. Histopathology confirmed the tumor had oval and spindle shaped fibroblastic cells with rich collagen deposition. It stained positive for ER, CD34, desmin and CD10 but negative for -smooth muscle actin and S-100. A mammary-type myofibroblastoma was diagnosed based on these results.

**Discussion:**

An extra-mammary myofibroblastoma is very rare and first reported in 2001. Since then, over 160 cases have been reported. On immunohistochemistry, these lesions are characteristically positive for CD34 and desmin, with variable staining for α-smooth muscle actin. Once the diagnosis is made, regardless of size or location, this tumour behaves in a benign fashion after surgical excision reported so far.

**Conclusion:**

This case is rare, but the correct diagnosis and treatment is important for good prognosis.

## Highlights

• Mammary-type Myofibroblastoma of the Inguinal Region is very rare.
• Prognosis of this disease is good after surgical resection.
• The correct diagnosis and treatment is important.
• We think that it is useful to share knowledge widely about this disease.

## 1 Introduction

Mammary-type myofibroblastoma occurring outside the breast is a rare, benign neoplasm histologically identical to mammary myofibroblastoma, a similarly benign breast tumour. First described in 2001 [1], in a series of 9 cases, these tumours have become recognised but remain rare. We present an additional case and a review of the literature. The work has been reported in line with the SCARE criteria [2].

## 2 Presentation of case

**Fig. 1:**
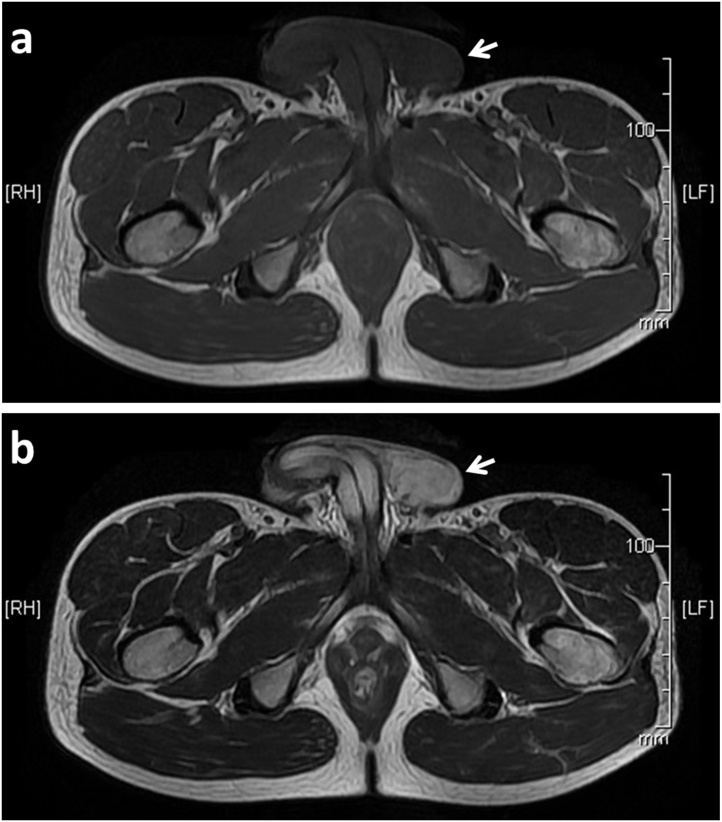
MRI showed a 50 mm low intensity well-demarcated lesion on T1-weighted image(a) and a high intensity lesion on T2-weighted image (b).

**Fig. 2:**
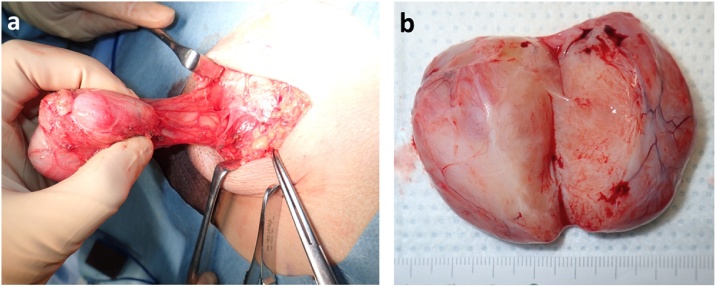
The cut surface was white, smooth, and glistening, without necrosis or hemorrhage.

**Fig. 3:**
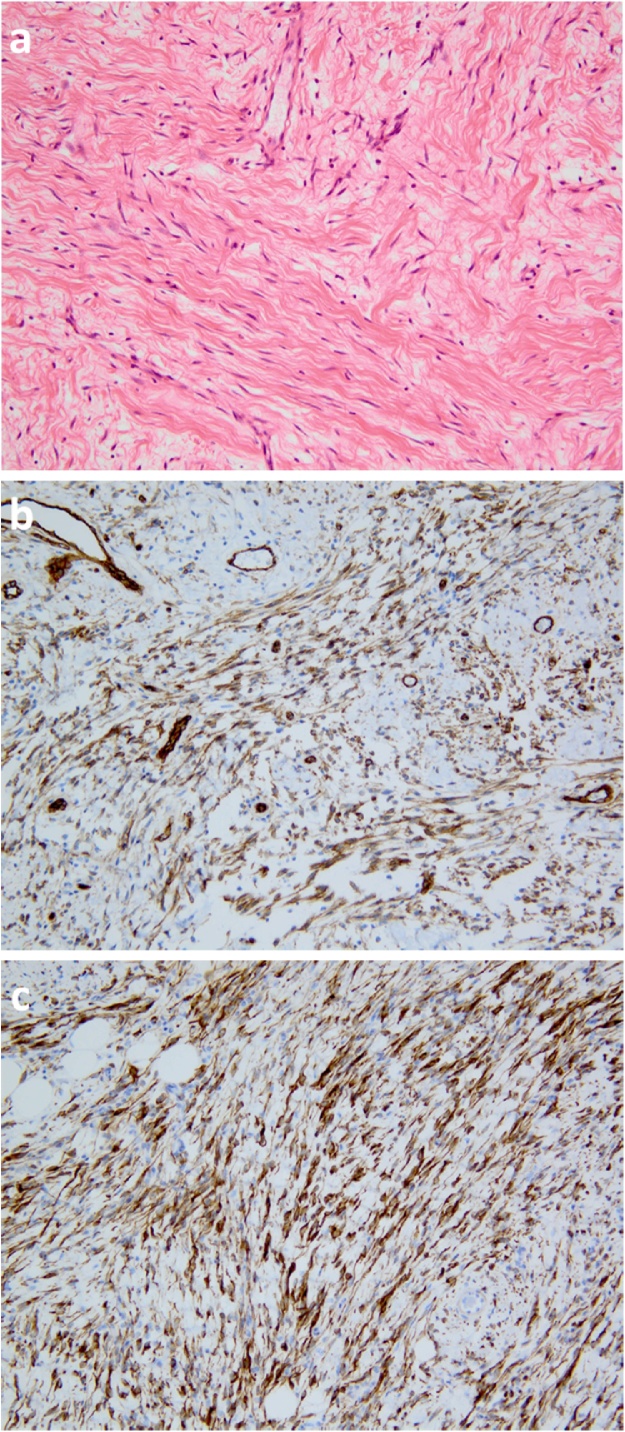
On histology, the lesion was characterized by oval or spindle fibroblastic cells (a). The tumor cells showed immunopositivity for CD34 (b) and desmin (c).

A 38 year old male was admitted to our hospital for evaluation of a left inguinal swelling which had been present and enlarging for 16 months. The tumour was 50 mm in size, well circumscribed, firm, fully mobile and painless. No inguinal or other local lymphadenopathy was present. Laboratory tests were normal. Ultrsonograph and CT revealed a well defined tumour with no communication to the spermatic cord. Magnetic resonance imaging showed that the tumor was T1 low and T2 high indicating a low fat content (Fig. 1a, b). These investigations could not provide a definitive diagnosis. Trans-inguinal surgical excision confirmed 50 mm tumor located in the inguinal canal with a clear surface and a thin capsule (Fig. 2a). No communication between the tumor and the spermatic cord or testis was found. The cut surface of the tumor showed a white uniform appearance. Necrosis, hemorrhage and cystic lesion was not noted (Fig. 2b). Histopathology reported the tumor had oval and spindle shaped fibroblastic cells with rich collagen deposition (Fig. 3a). Staining for ER, CD34 (Fig. 3b), desmin (Fig. 3c), and CD10 was positive, but α-smooth muscle actin and S-100 was negative. Mammary-type myofibroblastoma was diagnosed based on these results. The postoperative course was uneventful and the patient is doing well with no recurrence of the tumor at 3 years.

## 3 Discussion

Myofibroblastoma was first described in 1987 as a benign, soft tissue tumor of the breast [3]. The most common presentation is a painless, slowly growing mass. The tumor is characteristically well circumscribed, composed of uniform spindle cells. It is considered entirely benign and surgical removal is curative [3]. On immunohistochemistry, these lesions are characteristically positive for CD34 and desmin, with variable staining for smooth muscle actin [4]. An extra-mammary location of a myofibroblastoma is very rare and first reported in 2001 [1]. Since then, over 160 cases have been recorded [5]. The mean age of diagnosis is 52.5 years-old with a male predominance [3,6]. The reason for extra-mammary location is not resolved. However, it usually arises along the embryonic milk-line, which extends from the mid-axilla to medial groin [1]. This possible ectopic breast tissue is one possible explanation but several cases outside of this embryonic line have been also reported [1,[7], [8], [9], [10]]. The most common anatomic site of these tumors was the inguinal region [6,7].

Multi-modalities such as US, CT, and MRI cannot diagnose the condition and it is rarely possible to have a correct diagnosis before surgery. Needle biopsy or intraoperative frozen section diagnosis are possible and important methods to establish a diagnosis but nearly all cases make a final diagnosis following surgical excision [6,[10], [11], [12], [13]]. On histopathology, this tumour can be confused with spindle cell lipoma and other differential diagnoses include cellular angiofibroma, angiomyofibroblastoma, soft tissue perineurioma, nodular fasciitis, and malignant tumors such as spindle cell liposarcoma and malignant peripheral nerve sheath tumors [1]. Differentiating mammary-type myofibroblastoma from these, especially from spindle cell lipoma, is often difficult, and depends on the immunohistochemical findings. Mammary-type myofibroblastoma and spindle cell lipoma are benign, spindle cell neoplasms that show immunoreactivity with CD34 [14,15]. Subtle histological differences between the lesions do exist, as mammary-type myofibroblastoma has less fat than spindle cell lipoma and contain a more prominent, hyalinized stroma. In addition, mammary-type myofibroblastoma shows desmin positivity on immunohistochemistry, while spindle cell lipoma does not [1]. The sensitivity of CD34 and desmin for detecting mammary-type myofibroblastoma is close to 90% [5]. Once the diagnosis is made, the prognosis of this disease is good [3]. Regardless of size or location, these lesions behaved in a benign fashion after surgical excision reported so far. Out of the cohort of 143 reported cases only one had a local recurrence 20 years after initial excision. In the 8 cases with positive surgical resection margins, there has been no reported recurrence [5]. Our patient is well without recurrence of the tumor after 3 years.

## 4 Conclusion

Extra-mammary-type myofibroblastoma is a rare, benign soft tissue neoplasm with no known malignant behavior and excellent prognosis following surgical excision. Being rare, the correct diagnosis can be difficult, and to achieve this, the accumulation of cases is needed.

## Conflict of interest

The authors declare there is no conflict of interest.

## Funding source

There are no sponsors involved in the case report.

## Ethical approval

This case report was exempt from ethical approval in our institution.

## Consent

We have parental consent on behalf of the patient for publication of the submitted article and images.

## Author contribution

Atushi Ishihara operated on the patient, and performed the background research, writing of the manuscript, and submission of the manuscript. He has no conflicts of interest. Takeo Yasuda operated on the patient, and performed the editing of the manuscript. He has no conflicts of interest. Yukari Sakae, Masayuki Sakae, Tooru Hamada, and Takashi Tsukazaki contributed to editing the manuscript. They have no conflicts of interest. Hideki Tshkazaki operated on the patient. He has no conflicts of interest. Masaru Furumoto contributed to make a correct diagnosis and editing the manuscript. He has no conflicts of interest. All authors have made a significant contribution and arrowed the final version of the manuscript for publication.

## Registration of research studies

Not applicable.

## Guarantor

Takeo Yasuda.

## Provenance and peer review

Not commissioned, externally peer-review.
